# Mapping Topsoil Total Nitrogen Using Random Forest and Modified Regression Kriging in Agricultural Areas of Central China

**DOI:** 10.3390/plants12071464

**Published:** 2023-03-27

**Authors:** Liyuan Zhang, Zhenfu Wu, Xiaomei Sun, Junying Yan, Yueqi Sun, Peijia Liu, Jie Chen

**Affiliations:** 1 School of Agricultural Sciences, Zhengzhou University, Zhengzhou 450001, China; 2 Henan Provincial Station of Soil and Fertilizer, Zhengzhou 450002, China; 3 School of Politics and Public Administration, Zhengzhou University, Zhengzhou 450001, China; 4 Henan Academy of Geology, Zhengzhou 450001, China; 5 Contemporary Capitalism Research Center, Zhengzhou University, Zhengzhou 450001, China

**Keywords:** topsoil, total nitrogen, random forest, modified regression kriging, digital soil mapping, Henan province, China

## Abstract

Accurate understanding of spatial distribution and variability of soil total nitrogen (TN) is critical for the site-specific nitrogen management. Based on 4337 newly obtained soil observations and 33 covariates, this study applied the random forest (RF) algorithm and modified regression kriging (RF combined with residual kriging: RFK, hereafter) model to spatially predict and map topsoil TN content in agricultural areas of Henan Province, central China. According to the RFK prediction, topsoil TN content ranged from 0.52 to 1.81 g kg^−1^, and the farmland with the topsoil TN contents of 1.00–1.23 g kg^−1^ and 0.80–1.23 g kg^−1^ accounted for 48.2% and 81.2% of the total farmland area, respectively. Spatially, the topsoil TN in the study area was generally higher in the west and lower in the east. By using the Boruta variable selection algorithm, soil organic matter (SOM) and available potassium contents in topsoil, nitrogen deposition, average annual precipitation, livestock discharges, and topsoil pH were identified as the main factors driving the spatial distribution and variation of soil TN in the study area. The RF and RFK models used showed the expected performance and achieved acceptable TN prediction accuracy. In comparison, RFK performed slightly better than the RF model. The R^2^ and RMSE achieved by the RFK model were improved by 4.5% and 4.5%, respectively, compared with that by the RF model. However, the results suggest that RFK was inferior to the RF model in quantifying prediction uncertainty and thus may have a slight disadvantage in model reliability.

## 1. Introduction

Soil total nitrogen (TN) is one of the most important indicators of soil productivity and the biogeochemical cycle, and plays an essential role in agroecosystem functioning and climate change mitigation [1,2,3,4]. Low soil TN content suggests that nitrogen may become a crucial limiting factor for primary productivity in agroecosystems, while excessive soil TN content implies the risk of agricultural non-point source pollution and greenhouse gas emissions [5,6,7,8,9,10]. Spatially predicting the distribution and variability of soil TN and determining its main controlling factors are of great significance for understanding the carbon–nitrogen cycle in agroecosystems, implementing site-specific nitrogen management, and maintaining nitrogen dynamic balance at regional, landscape, and field scales, which help improve soil quality, increase food production, prevent agricultural non-point source pollution, and reduce greenhouse gas emissions [7,11,12,13].

It is well known that soil TN content, especially in arable soils, is not only affected by natural factors such as topography, parent material, climate, and biology, but also by anthropic activities such as fertilization, irrigation, crop rotation, tillage, and straw management, etc. The heterogeneity in time and space of the above mentioned factors leads to great variability of soil TN, making it one of the most challenging soil properties to predict and manage [11,14,15]. The technical approach most commonly used to address the spatial distribution and variation of soil TN content is digital soil mapping (DSM) [16,17,18,19], which overcomes the disadvantages of costly and time-consuming conventional mapping, especially on a large regional scale [20]. The DSM technology is based on the soil–landscape model, which is a map of soil properties by fitting quantitative relationships between measured soil properties and environmental covariates, and applying spatial analysis and mathematical methods to predict the spatial distribution of soils [21]. Over the past two decades, machine learning (ML) algorithms have increasingly been used as DSM tools for soil spatial prediction, largely due to the increased availability of open access data and the dramatic growth in computer power [22,23,24]. Briefly, for regions with sparse sample point information, machine learning can predict soil properties (e.g., soil TN) for the whole region by learning the relationship between environmental and target variables [25], without prior statistical assumptions [26,27,28]. Among ML models, the tree-based algorithms represented by random forest (RF) have shown the best performance and gained the most popularity in predicting soil properties [29,30,31,32,33].

However, the ML approaches, including RF, only quantitatively fit the complex and nonlinear deterministic relationships between soil observations and environmental covariates, ignore the spatial autocorrelation of soil observations, thus leading to the limitation of their prediction performance [34,35,36]. To address this shortcoming of ML approaches, Keskin and Grunwald [26] proposed the novel modified regression kriging (RK) methods, a hybrid model called the regression kriging type C, and investigated the deterministic component of soil variation using ML algorithms, which dealt with the stochastic part of variation by kriging interpolation of ML prediction residuals [34,37]. In most studies, these modified RK hybrid models significantly outperformed the corresponding standalone ML counterparts [32,38,39,40]. However, in a few cases, the prediction accuracy achieved by the hybrid models were no better or even worse than that achieved by ML algorithms [34,41,42,43]. So far, there is still no reasonable explanation for this conflicting conclusion.

The RF algorithm, a representative ML technique, and its hybrid model counterpart (RF combing with residual kriging: RFK, hereafter) were selected to spatially predict the topsoil TN in the agricultural area of Henan Province, central China. The objectives for this study were to (1) determine the spatial distribution, variability, and controlling factors of topsoil TN, (2) compare prediction performance of the RF and RFK models and analyze the differences in their performance, and (3) find their difference in quantitatively evaluating prediction uncertainty.

## 2. Results

### 2.1. Descriptive Statistics of Soil Total Nitrogen Observations

Summary statistics of the topsoil TN contents observed in the agricultural areas of the study area are presented in Table 1. The observed topsoil TN content ranged from 0.16 to 2.11 g kg^−1^, with a mean of 1.06 g kg^−1^. The coefficient of variation (CV) of the entire sample set was 27.00%, indicative of a moderate variability. Smaller kurtosis and skewness values indicate that the dataset was close to a normal distribution with a slight right (positive) skewness. There was no significant difference in the statistical characteristics of the entire set, calibration set, and validation set, indicating that all were well representative.

### 2.2. Relative Importance of Covariates

**Figure 1:**
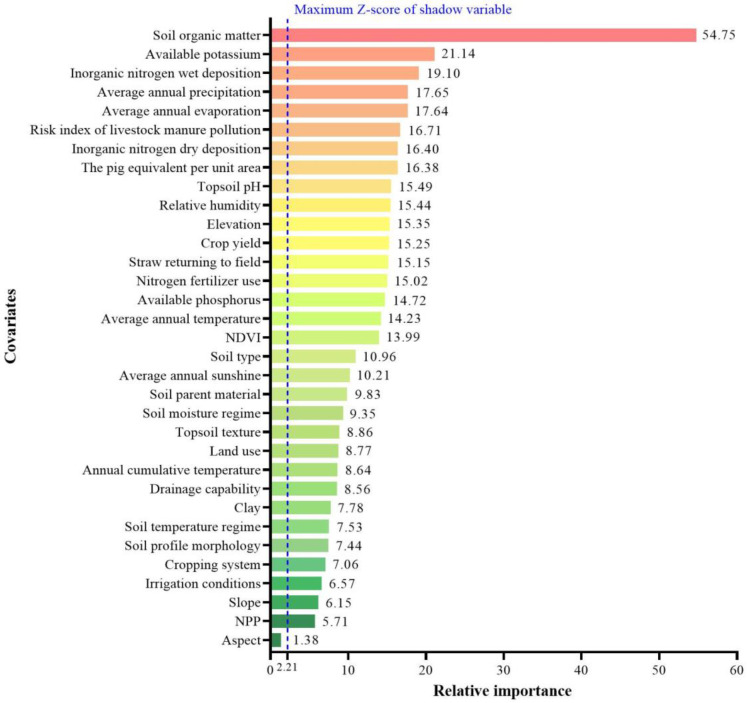
Relative importance of covariates.

Boruta’s quantitative evaluation showed that, except for aspect in the topographic attribute category, the relative importance of all the remaining 32 covariates was greater than the maximum value of the shadow variables (maximum *Z*-score), that is, they had an important influence on the spatial prediction of topsoil TN in the study area, and were involved in modeling as predictors. As shown in Figure 1, in addition to soil organic matter (SOM), which ranked first in the relative importance list by absolute dominance, the covariates associated with soil nitrogen sources (e.g., application of livestock manure and N- fertilizer, atmospheric N-deposition), soil nutrient-holding capacity (e.g., available K and P contents), and the climatic covariates closely related to soil water availability (e.g., evaporation, precipitation, and relative humidity) ranked higher (Figure 1).

### 2.3. Spatial Distribution and Variability of Topsoil TN

**Figure 2:**
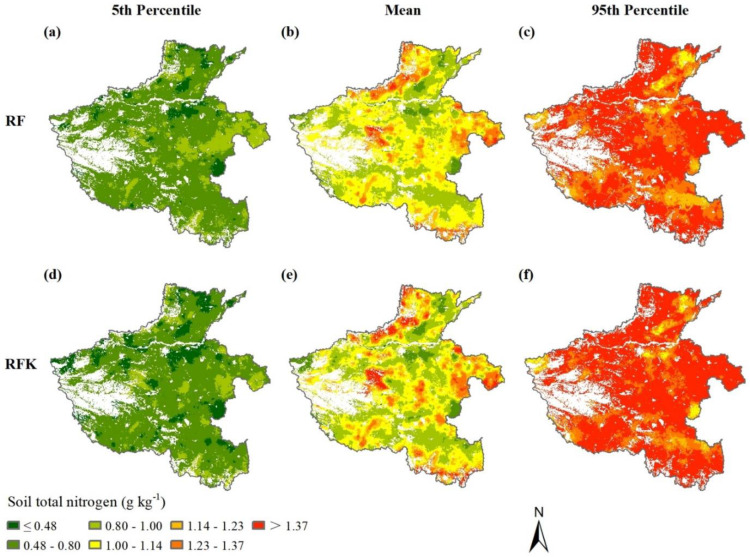
Lower limit (**a**), mean (**b**) and upper limit (**c**) of topsoil TN predicted by RF; lower limit (**d**), mean (**e**) and upper limit (**f**) of topsoil TN predicted by RFK.

Based on the covariate set established by the variable selection using the Boruta algorithm, the spatial distribution of topsoil TN content predicted by the RF model was shown in Figure 2b. As tested, the residues from the RF prediction had spatial autocorrelation (Moran’s I = −0.06, Z-score = −7.06, *p* < 0.01) and matched the normal distribution (K-S test *p* > 0.05). The optimal semi-variance model parameters are shown in Table 2. The results showed that the best-fitting model for the RF residuals was an exponential model. The nugget and sill values were 0.0018 and 0.0447, respectively. The nugget effect was 4.02%, indicating that the RF residuals exhibited strong spatial dependence. Then, the spatial distribution of topsoil TN residues was estimated by OK interpolation. The final TN prediction by the RFK model was generated by adding the deterministic component from the RF model with the residual interpolation (Figure 2e). According to the RFK prediction, the topsoil TN content in the study area ranged from 0.52 to 1.81 g kg^−1^, with a mean of 1.06 g kg^−1^. Compared with the TN observations in the calibration set, the distribution range of predicted TN content was significantly narrowed, reflecting the apparent smoothing effect of the RFK prediction. The agricultural lands with topsoil TN content of 1.00–1.23 g kg^−1^ were the most widely distributed in the study area, accounting for 48.2% of the total agricultural area, followed by the lands with TN content of 0.80–1.00 g kg^−1^, covering 33.0% of the total agricultural area. The agricultural lands with topsoil TN > 1.37 g kg^−1^ were mainly distributed in the mountainous areas of western Henan Province, while the lands with topsoil TN contents ≤0.48 g kg^−1^ were concentrated in the Huang–Huai–Hai plain within the study area. Spatially, the topsoil TN in the study area showed considerable spatial variability.

### 2.4. Comparison of Model Performance

**Figure 3:**
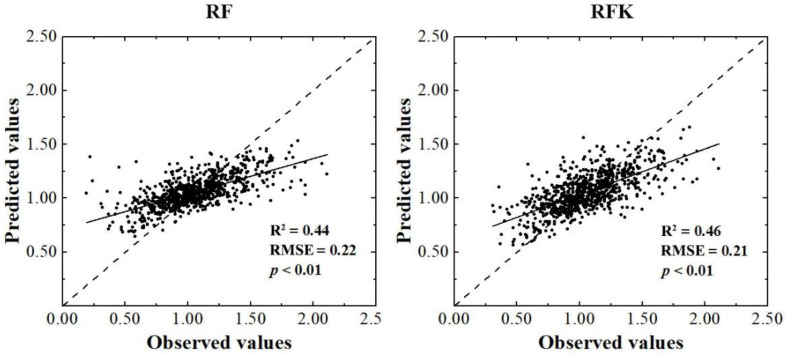
Predictive performance comparison between RF and RFK models.

The independent validation showed that in predicting topsoil TN content in the study area (Figure 3), the R^2^ achieved by the RF and RFK models was 0.44 and 0.46, and the RMSE was 0.22 and 0.21, respectively. The RFK model outperformed the RF model in terms of predictive performance. Based on the calculation of the CI width, the uncertainty of topsoil TN predictions were quantitatively evaluated by counting the percentage of topsoil TN observations that fell within the specified 90% CI, according to the technical specifications of GlobalSoilMap [44,45] (Table 3). Approximately 92.4% of the topsoil TN observations in the validation set fell into the 90% CI of the RF model, demonstrating an acceptable reliability of the predictions. In comparison, the CI coverage probability of RFK model was higher than that of the RF model, and the percentage of soil observations in the validation set falling into 90% CI was 98.2%, significantly deviating from the theoretical range, indicating that the uncertainty of model prediction was overestimated.

## 3. Discussion

### 3.1. Covariate Contributions

The relative importance of the covariates derived from the variable selection algorithm refers to the relative influence of the covariates on the spatial prediction of the target soil variables. If the model prediction was reliable, then the relative importance of the covariates largely implied the ability of the covariates to drive the spatial distribution and variation of the target soil variables. Therefore, although the RF used in this study was not an explanatory model, it could reveal the driving factors of spatial distribution and variation of topsoil TN content in the study area from the relative importance of covariates involved in modeling.

**Figure 4:**
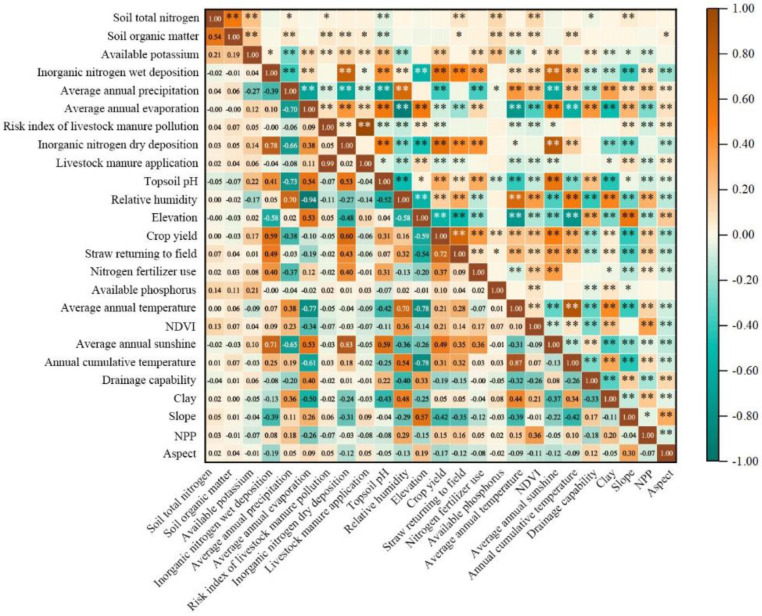
Pearson correlations between topsoil TN and covariates, * and ** denote significance levels of *p* < 0.05 and *p* < 0.01, respectively.

**Figure 5:**
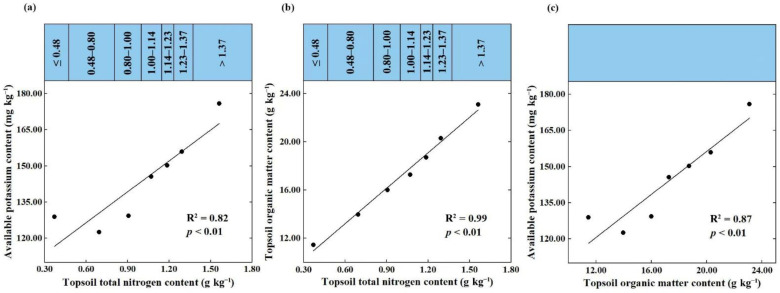
Scatterplots of topsoil TN against available potassium (**a**) and SOM (**b**), of topsoil SOM against available potassium (**c**), according to the average content of each grade.

As expected, SOM content dominated the spatially explicit estimation of the topsoil TN in the study area, which was consistent with most other studies [12,16,46,47,48]. The statistics of soil observations in the calibration set showed that the content of SOM and topsoil TN was positively correlated at the *p* < 0.01 level (Figure 4), which indicated that most topsoil TN existed in organic form, and perhaps some inorganic nitrogen was adsorbed on the SOM functional groups. The relative importance of available potassium content in topsoil ranked second among all covariates, which may be due to two causes. First, the widespread use of compound fertilizer containing N, P, K elements increased the possibility of the coexistence of available nitrogen and available potassium. Secondly, and most importantly, there was a close correlation between available potassium and TN content. If the topsoil TN content in the calibration set is divided into seven grades according to the legend grading standard in Figure 2, and the scatterplot of topsoil TN against available potassium is made according to the average content of each grade, then there is an almost perfect linear correlation between them (Figure 5a). This was most likely attributed to the fact that the available potassium in soil was mainly adsorbed to organic colloid in the form of exchangeable cations. Such a perfect correlation also existed between SOM and topsoil TN (Figure 5b), and available potassium (Figure 5c).

Both forms of N deposition ranked third and seventh, respectively in the relative importance of covariates, indicating that they played a very important role in the topsoil TN prediction, which has rarely been reported in other soil TN estimation studies conducted in China [18,33,47,48]. In fact, few studies have included N deposition as a covariate for soil TN prediction, possibly because the intensity of N deposition has shown a dramatic decline in most parts of the country over the past two decades. Nevertheless, at least in this study area, N deposition seemed to remain an important source of soil nitrogen, and had a significant contribution to topsoil TN content. The relative importance of average annual precipitation ranked fourth among the covariates, and had a significantly positive correlation with topsoil TN at the *p* < 0.05 level in the calibration set (Figure 4), which was consistent with the identification of influencing factors of soil TN in other studies [33,48,49,50]. We believe that precipitation, together with evaporation, has a dual impact on soil TN: one was to affect SOM accumulation and thus TN content; the other was to alter soil water availability to drive nitrogen behavior, such as leaching and volatilizing.

Two covariates characterizing the potential nitrogen output of the local livestock industry, namely the pig equivalent per unit area and the risk index of livestock manure pollution, were also relatively high in the relative importance ranking, among which the risk index of livestock manure pollution was significantly and positively correlated with the topsoil TN content at the *p* < 0.05 level. In previous studies of soil TN prediction, almost none included the livestock-related data layer as a covariate, possibly due to the small size of the livestock industry in these study areas. In Henan province, however, the comprehensive production capacity of animal husbandry has been continuously enhanced over the past decade. In 2021, the output value of animal husbandry in the province ranked the second in the country, accounting for 28.7% of the total agricultural output value of the province. The impact of livestock waste discharge on soil nitrogen should not be ignored.

Topsoil pH also ranked in the top 10 covariates, and was significantly negatively correlated with topsoil TN at the *p* < 0.01 level. This correlation has also been found by previous studies [47]. Many studies have shown that the entry of exogenous nitrogen, such as N fertilizer application and N deposition, could increase the SOM and soil TN contents while leading to the decrease in soil pH [47,51,52,53,54].

### 3.2. Prediction Accuracy

As mentioned above, soil TN is one of the most difficult soil attributes to be spatially predicted due to the high diversity and great spatial–temporal variability of influencing factors. Given that the study area covered 167,000 km^2^, the performance of the RF and RFK models used in this study and the achieved accuracy of topsoil TN prediction were in line with expectations. Under the conditions of comparable soil sample density, covariate availability, and landscape complexity, the smaller the geographical scope of the study area, the better the prediction performance of the model used. Liu et al. [55] successfully predicted soil TN content using a multiple linear regression (MLR) model in a small watershed of 4.2 km^2^ in Shandong Province, China, and achieved a prediction R^2^ of 0.69. In the study conducted by Wadoux et al. [56] in the metropolitan territory of France covering about 540,000 km^2^, based on the soil observations from the LUCAS dataset, the topsoil TN prediction using the RF model just obtained an R^2^ of 0.20, while the RMSE was as high as 1.52. In Zhejiang Province (located in East China and with a total area of 104,300 km^2^), Deng et al. [47] used the RF model to spatially predict topsoil TN content and achieved an R^2^ of 0.65. The density of the soil observations in Deng et al.’s study was about seven times that of our soil observations. We believed that the much higher soil observation density might be one of the key reasons for the significantly higher R^2^. However, the prediction RMSE achieved by Deng et al.’s study was 0.45, much higher than the 0.22 obtained in this study. Considering the differences in topsoil TN levels between the two study areas, the normalized RMSE (NRMSE) was calculated by dividing the RMSE by the mean of the TN observations. It was found that the NRMSE of topsoil TN prediction in Zhejiang study area was 0.25, while that in our study area was 0.20. It seems that increasing the sample observations could significantly promote the model capacity to explain the spatial variation of topsoil TN, but it might not effectively reduce the prediction deviation.

In terms of R^2^ and RMSE, the accuracy of the RFK model was better than the RF model for topsoil TN prediction in the study area. The R^2^ and RMSE obtained by the RFK model improved by 4.5% and 4.5%, respectively, compared with those obtained by the RF model. The superiority of RFK over the RF model is visually demonstrated by the plots of predicted against measured values of the topsoil TN contents (Figure 3). As shown in Figure 3, although both models display a similar pattern, RFK scatter is less tight around the 1:1 line, and overestimated lower and underestimated higher TN content values to a lesser extent than RF. This finding was close to the studies conducted by Takoutsing and Heuvelink [37].

Many studies have reported that the RK model and its modified visions were superior to competitors to varying degrees in spatially predicting soil TN [18,48,57,58]. In comparison, the performance advantage of RFK over RF in this study was smaller than in most previous studies. First, the relatively large study area increased the terrain diversity, landscape complexity and the soil heterogeneity, leading to the decrease in effective control scope of the spatial autocorrelation of soil TN [26,59]. Therefore, the existing soil observations were not enough to predict the spatial stochastic variation of soil TN well. With the same calibration dataset, using the OK model to predict the topsoil TN in this study area, the achieved R^2^ was only 0.21, but the RMSE was as high as 0.25. Obviously, the performance of OK was inferior to the RF and RFK models. Second, the model structure and the used covariates largely influenced the residual spatial autocorrelation of deterministic prediction. The RF used in this study was a tree-based ML model populated with all relevant variables, which usually leaves no or weak residual spatial autocorrelation [26]. Thus, the substantial superiority of RFK performance could not achieved by OK of the residuals from the RF model.

### 3.3. Prediction Uncertainty

One of the main advantages of the DSM approach is that it allows for quantitative analysis of prediction uncertainties. Based on the statistical results of the validation sample points (Table 3), the CI of RF (92.40%) is closer to the theoretical value of 90% compared to RFK (CI of 98.21%), indicating that RF outperforms RFRK in terms of quantitative estimation of spatial prediction uncertainty. Similarly, Takoutsing and Heuvelink [37] found in a recent study at the landscape scale that regression kriging (RK) was better at predicting a variety of soil properties by achieving lower RMSE values, but worse at quantifying prediction uncertainty than the RF model. In this study, the results showed that the performance of the RK and RF models did not change in terms of both prediction accuracy and quantification of prediction uncertainty when the trend term in the RK was fitted with the RF model instead of the regression model.

## 4. Materials and Methods

### 4.1. Study Area

**Figure 6:**
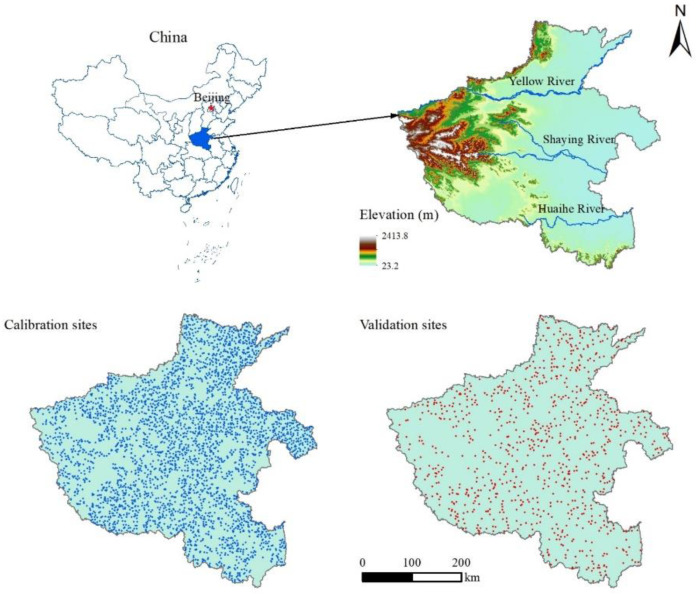
Geographical location of the study area and spatial distribution of the soil sampling sites.

Henan province (31°23′–36°22′ N and 110°21′–116°39′ E) is located in the middle and lower reaches of the Yellow River in central China (Figure 6), covering a total land area of 167,000 square kilometers, of which 7.51 million hectares are arable land. Henan Province is generally high in the west and low in the east, with an altitude range of 23.2–2413.8 m. The province has a variety of landforms, among which mountains and hills account for 44.3% and plains and basins account for 55.7% of the total land area. Most of the province is in the warm temperate zone, belongs to a continental monsoon climate with a transition from the northern subtropical zone to the warm temperate zone, and features four distinct seasons and simultaneous rain and heat. The average annual temperature of the province from south to north is 10.5–16.7 °C, the average annual precipitation is 464.2–1193.2 mm, the most rainfall is from June to August, the average annual sunshine is 1285.7–2292.9 h, and the annual frost-free period is 208.7–290.2 days, which is suitable for a wide range of crops. The cropping system in Henan Province mainly adopts a winter wheat–summer maize (northern region) and a rice–winter wheat (southern region) crop rotation. As a major agricultural province, grain production in Henan Province plays an important role in China’s food security strategy. In 2022, the grain output of the province reached 67.89 billion kg, ranking the second in China, and exceeding 50 billion kg for 16 consecutive years and 65 billion kg for the sixth consecutive year. According to the Chinese Soil Taxonomy, the types of major agricultural soils in Henan Province consist of several suborders of Cambosols (WRB: Vertic Cambisols, Calcaric Cambisols), Argosols (WRB: Calcic Luvisols, Haplic Luvisols) and Primosols (WRB: Fluvic Cambisols, Calcaric Fluvisols), and Stagnic Anthrosols (WRB: Hydragric Anthrosols).

### 4.2. Soil Sampling and Measurement

For the purpose of monitoring cultivated land quality and promoting formulated fertilization, a total of 4337 topsoil samples were collected in the agricultural areas of Henan Province from 2017 to 2019. Taking the data layers of topography, land use and soil type as the basic strata, the soil sample sites were generated through a stratified random strategy and located using a global positioning system (GPS). At each location, the topsoil sample was taken at a depth of 0–20 cm, which weighed about 1 kg and was composed of the subsamples gathered from the corners and center of a 20 × 20 m quadrat. All the soil samples were carefully packed into cotton bags, labeled, and transported to the laboratory. After air-drying at room temperature for three weeks, the soil samples were removed from plant roots, litter, stones, and alien items, and sieved with a 0.25 mm mesh of stainless steel. The soil TN content was measured using an automatic Kjeldahl analyzer and the laboratory operations followed the relevant technical regulations in Agricultural Industry Standards of the People’s Republic of China No. NY/T1121.

The soil samples (n = 4337) were split into calibration (n = 3470, 80%) and validation (n = 867, 20%) sets using the createDataPartition function in the caret package [60] in R 4.0.3 [61]. The calibration set was used to train the RF and RFK models, while the validation set was prepared for independent validation. The spatial distribution of soil sampling sites in calibration and validation sets is shown in Figure 1.

### 4.3. Covariates and Variable Selection

A total of 33 covariates that had pedogenetic associations with soil nitrogen or explanatory capacity for soil nitrogen behavior were collected and prepared as potential predictors of topsoil TN content. These 33 covariates could be roughly regarded as six categories, namely, nitrogen sources, soil properties, topographic attributes, climate characteristics, organism features, and management practices. Nitrogen fertilizer use, atmospheric nitrogen deposition, the pig equivalent per unit area, and straw returning to field were classified into the category of nitrogen sources. Soil property category included organic matter, available phosphorus and available potassium contents in topsoil, soil type, soil parent material, soil profile morphology, topsoil pH, topsoil texture, topsoil clay content, soil temperature regime, and soil moisture regime. Terrain attribute category mainly comprised elevation, slope and aspect. The climate characteristics category included average annual temperature, average annual precipitation, average annual evaporation, relative humidity, average annual sunshine, and annual cumulative temperature. The organism features included the normalized difference vegetation index (NDVI), net primary productivity index (NPP), and crop yield. The management practices category was composed of land use, cropping system, irrigation condition, drainage capability, and risk index of manure pollution. The brief descriptions of 33 covariates and their sources were listed in Table 4. To achieve the uniformity of spatial reference and resolution, all covariates were converted to WGS1984_UTM_49N projection coordinates and resampled to 1000 m resolution in ArcGIS 10.7.

For the vast majority of ML models, the prediction accuracy does not entirely depend on the number of covariates involved in modeling. Redundant, irrelevant covariates usually have a negative impact on the model performance. Variable selection, or feature selection, thus becomes an important aspect of model building and helps in building predictive models free from correlated variables, biases, and unwanted noise [34,62]. In this study, Boruta, an algorithm as a wrapper around RF, was chosen to conduct variable selection and valuation of covariate relative importance on the R statistical computing and analysis platform [63].

### 4.4. Predictive Models

The RF algorithm is a typical bagging algorithm (bootstrap aggregation) in ensemble learning [64]. It contains a number of decision trees and uses bootstrap resampling methods to perform put-back sampling of the dataset to train each decision tree in the model. Finally, the results of each tree are integrated. To generate a predictive model, the RF algorithm needs two user-defined parameters to be set, namely the number of trees to grow in the forest (*ntree*) and the number of covariates selected at each split (*mtry*). Many cases have demonstrated that 150 trees were sufficient to generate stable outcomes [65,66]. In the present study, we fixed *ntree* = 200. By default, we settled *mtry* to the rounded down square root of the total number of covariates. This study carried out the RF modeling using the randomForest package [67] in R 4.0.3 and the final prediction of topsoil TN content was presented as the average value of all the tree predictions generated based on a bootstrap sample of the calibration set.

The residuals from the RF model were obtained by subtracting the predicted TN content from the measured TN content at the same site. Then, ordinary kriging (OK) was used to obtain the spatial distribution of the RF residuals, and finally the interpolated results of the RF residuals were added to the RF prediction results to obtain the RFK prediction results. TN prediction from the hybrid model RFK can be described as follows:

Ŷ_RFK(s)_ = Ŷ_RF(s)_+έ_OK(s)_ (1)

where Ŷ_RFK(s)_ is the predicted TN by the hybrid model RFK at the locations, Ŷ_RF(s)_ is the predicted TN by the RF model, and έ_OK(s)_ is the residual estimation by ordinary kriging (OK) interpolation. It should be emphasized that before fitting the semi-variance function, Spatial autocorrelation of RF residuals using the global Moran’s I index test according to the requirements of ordinary kriging for data. If there is spatial autocorrelation and the residuals of RF conform to a normal distribution, OK interpolation can be used. If there is no spatial autocorrelation, the predicted topsoil TN by RF will be the output result. In the present study, spatial autocorrelation analysis, semi-variance analysis, and OK interpolation of RF residuals were all implemented in the ArcGIS 10.7 environment [18,68].

### 4.5. Evaluation of Model Performance

An independent validation approach was applied to assess performance of the prediction models in spatially predicting topsoil TN. Two commonly used assessment metrics, namely, the root mean square error (RMSE) and the coefficient of determination (R^2^), were chosen to compare the accuracy of topsoil TN prediction by the RF and RFK models.

(2) $$\mathrm{RMSE}=\sqrt{\frac{1}{n}\overset{n}{\underset{i=1}{\sum }}{\left(o_{i}-p_{i}\right)}^{2}}$$

(3) $$R^{2}=1-\frac{\sum_{i=1}^{n}{\left(o_{i}-\;p_{i}\right)}^{2}}{\sum_{i=1}^{n}{\left(o_{i}-\;\bar{o}\right)}^{2}}$$

where *n* is the validation sample size, $o_{i}$ and $p_{i}$ represent the observed and predicted values, respectively, of topsoil TN content by a given method at the *i*th locations, and $\bar{o}$ is the average of the observed values of topsoil TN for the validation samples. Of the metrics used, RMSE summarizes the magnitude of the residuals, and a smaller RMSE indicates a higher accuracy of model prediction, while *R*^2^ indicates the proportion of the topsoil TN variance explained by the covariate set.

RMSE and R^2^ can evaluate the accuracy of a model, but they lack the ability to quantify the uncertainty of the model. In this study, the 5% and 95% quantiles of the quantile regression forest (QRF) [69] prediction were regarded as the lower and upper limits of the 90% confidence interval (CI) width of the RF model, respectively. Assuming that the kriging interpolation of deterministic residuals followed the normal distribution, the upper and lower limits of 90% CI of the residual kriging was calculated at μ ± 1.645σ, where μ and σ were the mean and standard deviation of the predicted residuals, respectively [44]. Then, the 90% CI width of RFK model can be jointly determined by the upper and lower limits of 90% CI of RF model and kriging interpolation. Finally, we calculated the percentage of topsoil TN observations that fell at 90% CI to evaluate the ability of RF and RFK to quantify the uncertainty in spatial predictions of total soil nitrogen.

## 5. Conclusions

Under the combined effect of SOM, available potassium contents, nitrogen deposition, average annual precipitation, livestock discharges and topsoil pH, the TN content of agricultural soils in central China ranged from 0.52 to 1.81 g kg^−1^. The agricultural land with topsoil TN content between 1.00 g kg^−1^ and 1.23 g kg^−1^ was the most widely distributed, accounting for approximately half of the total agricultural land area. The spatial variability of topsoil TN in the study area was significant, and was overall high in the west and low in the east. Accurately predicting the spatial distribution of soil TN on a regional scale and understanding the drivers of soil TN provides the basis and technical support for site-specific nitrogen management and dynamic change control. In terms of R^2^ and RMSE achieved, RFK slightly outperformed the RF model. However, RFK was inferior to the RF model in quantifying prediction uncertainty. Overall, model performance evaluation should not be limited to the commonly used accuracy metrics, but should also consider the uncertainty of the quantitative prediction results.

## Figures and Tables

**Table 1:** Descriptive statistics of topsoil TN observations in the study area.

	Simple Size (n)	Mean (g kg^−1^)	Maximum (g kg^−1^)	Minimum (g kg^−1^)	SD ^1^ (g kg^−1^)	CV ^2^ (%)	Kurtosis	Skewness
Entire set	4337	1.06	2.11	0.16	0.28	27.00	0.64	0.36
Calibration set	3470	1.06	2.09	0.16	0.28	27.00	0.63	0.36
Validation set	867	1.06	2.11	0.19	0.28	27.00	0.69	0.34

^1^ standard deviation; ^2^ coefficient of variation.

**Table 2:** The optimal semi-variance model parameters for residuals from RF.

Variogram	Model	Nugget	Sill	Nugget/Sill (%)	R^2^	RSS ^1^	Range (km)
RF residuals	Exponential	0.0018	0.0447	4.02	0.345	9.325 × 10^−4^	2670

^1^ residual sum of squares.

**Table 3:** Percentages of topsoil TN observations in the validation set falling inside and outside the prescribed 90% CI.

	Inside	Outside	
		<5%	>95%
RF	92.40	3.60	4.00
RFK	98.21	0.49	1.30

**Table 4:** Brief description of the covariates in different categories.

Categories	Covariates	Data Source	Resolution/Scale
Nitrogen sources	Nitrogen fertilizer use	Field investigation during the soil sampling campaign	30 m
	Nitrogen wet deposition	National Science and Technology Infrastructure (http://rs.cern.ac.cn/index.jsp)	1000 m
	Nitrogen dry deposition	National Science and Technology Infrastructure (http://rs.cern.ac.cn/index.jsp)	10,000 m
	The pig equivalent per unit area	Field investigation during the soil sampling campaign	30 m
	Straw returning to field	Field investigation during the soil sampling campaign	30 m
Soil properties	Soil organic matter Available phosphorus	Henan Provincial Database for Cropland Quality Evaluation Henan Provincial Database for Cropland Quality Evaluation	1:200,000 1:200,000
	Available potassium	Henan Provincial Database for Cropland Quality Evaluation	1:200,000
	Topsoil pH	Henan Provincial Database for Cropland Quality Evaluation	1:200,000
	Soil type	Henan Provincial Database for Cropland Quality Evaluation	1:200,000
	Soil parent material	Henan Provincial Database for Cropland Quality Evaluation	1:200,000
	Soil profile morphology	Henan Provincial Database for Cropland Quality Evaluation	1:200,000
	Topsoil texture	Henan Provincial Database for Cropland Quality Evaluation	1:200,000
	Soil temperature regime	Soil Series of China, Volume Henan, 2019	1:200,000
	Soil moisture regime	Soil Series of China, Volume Henan, 2019	1:200,000
	Clay content	Henan Provincial Database for Cropland Quality Evaluation	1:200,000
Terrain attributes	Elevation	ASTER GDEM V3 30 m DEM (http://www.tuxingis.com/resource/aster_v3.html)	30 m
	Slope	Derived from ASTER GDEM V3 30 m DEM	30 m
	Aspect	Derived from ASTER GDEM V3 30 m DEM	30 m
Climate characteristics	Average annual temperature	National Meteorological Science Data Center (https://data.cma.cn/data/cdcdetail/dataCode/A.0012.0001.S011.html)	30 m
	Average annual precipitation	National Meteorological Science Data Center (https://data.cma.cn/data/cdcdetail/dataCode/A.0012.0001.S011.html)	30 m
	Average annual evaporation	National Meteorological Science Data Center (https://data.cma.cn/data/cdcdetail/dataCode/A.0012.0001.S011.html)	30 m
	Relative humidity	National Meteorological Science Data Center (https://data.cma.cn/data/cdcdetail/dataCode/A.0012.0001.S011.html)	30 m
	Average annual sunshine	National Meteorological Science Data Center (https://data.cma.cn/data/cdcdetail/dataCode/A.0012.0001.S011.html)	30 m
	Annual cumulative temperature	National Meteorological Science Data Center (https://data.cma.cn/data/cdcdetail/dataCode/A.0012.0001.S011.html)	30 m
Organism features	NDVI	China Resource and Environmental Science and Data Centre (http://www.resdc.cn.)	1000 m
	NPP	China Resource and Environmental Science and Data Centre (http://www.resdc.cn.)	1000 m
	Crop yield	Henan Provincial Database for Cropland Quality Evaluation	1:200,000
Management practices	Land use	Henan Provincial Database for Cropland Quality Evaluation	1:200,000
	Cropping system	Henan Provincial Database for Cropland Quality Evaluation	1:200,000
	Irrigation condition	Henan Provincial Database for Cropland Quality Evaluation	1:200,000
	Drainage capability	Henan Provincial Database for Cropland Quality Evaluation	1:200,000
	Risk index of livestock manure pollution	Field investigation during the soil sampling campaign	30 m

## Data Availability

Not applicable.

## References

[1] Bangroo S.A., Najar G.R., Achin E., Truong P.N. (2020). Application of predictor variables in spatial quantification of soil organic carbon and total nitrogen using regression kriging in the North Kashmir forest Himalayas. Catena.

[2] Zhang H., Shi L., Fu S. (2020). Effects of nitrogen deposition and increased precipitation on soil phosphorus dynamics in a temperate forest. Geoderma.

[3] Ma J., Cheng J., Wang J., Pan R., He F., Yan L., Xiao J. (2022). Rapid detection of total nitrogen content in soil based on hyperspectral technology. Inf. Process. Agric..

[4] Zhang X., Chen P., Dai S., Han Y. (2022). Analysis of non-point source nitrogen pollution in watersheds based on SWAT model. Ecol. Indic..

[5] Arabi M., Govindaraju R.S., Hantush M.M., Engel B.A. (2006). Role of watershed subdivision on modeling the effectiveness of best management practices with SWAT. JAWRA J. Am. Water Resour. Assoc..

[6] Guerrero A., De Neve S., Mouazen A.M., Sparks D.L. (2021). Chapter One—Current sensor technologies for in situ and on-line measurement of soil nitrogen for variable rate fertilization: A review. Advances in Agronomy.

[7] Li J., Chen L., Fu B., Zhang S., Li G. (2019). Spatial and temporal variation characteristics of non-point source N in surface water in Yuqiao reservoir basin. J. Geosci..

[8] Liao K., Lv L., Lai X., Zhu Q. (2021). Toward a framework for the multimodel ensemble prediction of soil nitrogen losses. Ecol. Model..

[9] Potarzycki J. (2011). Effect of magnesium or zinc supplementation at the background of nitrogen rate on nitrogen management by maize canopy cultivated in monoculture. Plant Soil Environ..

[10] Post W.M., Pastor J., Zinke P.J., Stangenberger A.G. (2021). Global patterns of soil nitrogen storage. Nature.

[11] Komolafe A.A., Olorunfemi I.E., Oloruntoba C., Akinluyi F.O. (2021). Spatial prediction of soil nutrients from soil, topography and environmental attributes in the northern part of Ekiti State, Nigeria. Remote Sens. Appl. Soc. Environ..

[12] Ma D., Zhang H., Song X., Xing S., Fan M., Heiling M., Liu L., Zhang L., Mao Y. (2022). Estimating soil organic carbon and nitrogen stock based on high-resolution soil databases in a subtropical agricultural area of China. Soil Tillage Res..

[13] Zhang Z., Hao M., Li Y., Shao Z., Yu Q., He Y., Gao P., Xu J., Dun X. (2022). Effects of vegetation and terrain changes on spatial heterogeneity of soil C–N–P in the coastal zone protected forests at northern China. J. Environ. Manag..

[14] Abebe G., Tsunekawa A., Haregeweyn N., Takeshi T., Wondie M., Adgo E., Masunaga T., Tsubo M., Ebabu K., Berihun M.L. (2020). Effects of land use and topographic position on soil organic carbon and Total nitrogen stocks in different agro-ecosystems of the upper Blue Nile Basin. Sustainability.

[15] Onwuka B., Mang B. (2018). Effects of soil temperature on some soil properties and plant growth. Adv. Plants Agric. Res..

[16] Dai L., Ge J., Wang L., Zhang Q., Liang T., Bolan N., Lischeid G., Rinklebe J. (2022). Influence of soil properties, topography, and land cover on soil organic carbon and total nitrogen concentration: A case study in Qinghai-Tibet plateau based on random forest regression and structural equation modeling. Sci. Total Environ..

[17] Sadayappan K., Kerins D., Shen C., Li L. (2022). Nitrate concentrations predominantly driven by human, climate, and soil properties in US rivers. Water Res..

[18] Wang Y., Xiao Z., Aurangzeib M., Zhang X., Zhang S. (2021). Effects of freeze-thaw cycles on the spatial distribution of soil total nitrogen using a geographically weighted regression kriging method. Sci. Total Environ..

[19] Zhou T., Geng Y., Chen J., Pan J., Haase D., Lausch A. (2020). High-resolution digital mapping of soil organic carbon and soil total nitrogen using DEM derivatives, Sentinel-1 and Sentinel-2 data based on machine learning algorithms. Sci. Total Environ..

[20] Mulder V.L., de Bruin S., Schaepman M.E., Mayr T.R. (2011). The use of remote sensing in soil and terrain mapping—A review. Geoderma.

[21] Kalambukattu J.G., Kumar S., Raj R.A. (2018). Digital soil mapping in a Himalayan watershed using remote sensing and terrain parameters employing artificial neural network model. Environ. Earth Sci..

[22] Arrouays D., Poggio L., Salazar Guerrero O.A., Mulder V.L. (2020). Digital soil mapping and GlobalSoilMap. Main advances and ways forward. Geoderma Reg..

[23] Padarian J., Minasny B., Mcbratney A.B. (2019). Using deep learning for digital soil mapping. Soil.

[24] Zeraatpisheh M., Jafari A., Bagheri Bodaghabadi M., Ayoubi S., Taghizadeh-Mehrjardi R., Toomanian N., Kerry R., Xu M. (2020). Conventional and digital soil mapping in Iran: Past, present, and future. Catena.

[25] Heung B., Ho H.C., Zhang J., Knudby A., Bulmer C.E., Schmidt M.G. (2016). An overview and comparison of machine-learning techniques for classification purposes in digital soil mapping. Geoderma.

[26] Keskin H., Grunwald S. (2018). Regression kriging as a workhorse in the digital soil mapper’s toolbox. Geoderma.

[27] Mirchooli F., Kiani-Harchegani M., Khaledi Darvishan A., Falahatkar S., Sadeghi S.H. (2020). Spatial distribution dependency of soil organic carbon content to important environmental variables. Ecol. Indic..

[28] Tajik S., Ayoubi S., Zeraatpisheh M. (2020). Digital mapping of soil organic carbon using ensemble learning model in Mollisols of Hyrcanian forests, northern Iran. Geoderma Reg..

[29] Gomes L.C., Faria R.M., de Souza E., Veloso G.V., Schaefer C.E.G.R., Filho E.I.F. (2019). Modelling and mapping soil organic carbon stocks in Brazil. Geoderma.

[30] Hengl T., Heuvelink G.B., Kempen B., Leenaars J.G., Walsh M.G., Shepherd K.D., Sila A., MacMillan R.A., Mendes de Jesus J., Tamene L. (2015). Mapping soil properties of Africa at 250 m resolution: Random forests significantly improve current predictions. PLoS ONE.

[31] Khaledian Y., Miller B.A. (2020). Selecting appropriate machine learning methods for digital soil mapping. Appl. Math. Model..

[32] Silatsa F.B.T., Yemefack M., Tabi F.O., Heuvelink G.B.M., Leenaars J.G.B. (2020). Assessing countrywide soil organic carbon stock using hybrid machine learning modelling and legacy soil data in Cameroon. Geoderma.

[33] Wang S., Jin X., Adhikari K., Li W., Yu M., Bian Z., Wang Q. (2018). Mapping total soil nitrogen from a site in northeastern China. Catena.

[34] Keskin H., Grunwald S., Harris W.G. (2019). Digital mapping of soil carbon fractions with machine learning. Geoderma.

[35] Odeh I.O.A., McBratney A.B., Chittleborough D.J. (1995). Further results on prediction of soil properties from terrain attributes: Heterotopic cokriging and regression-kriging. Geoderma.

[36] Szatmári G., Pirkó B., Koós S., Laborczi A., Bakacsi Z., Szabó J., Pásztor L. (2019). Spatio-temporal assessment of topsoil organic carbon stock change in Hungary. Soil Tillage Res..

[37] Takoutsing B., Heuvelink G.B.M. (2022). Comparing the prediction performance, uncertainty quantification and extrapolation potential of regression kriging and random forest while accounting for soil measurement errors. Geoderma.

[38] Pouladi N., Møller A.B., Tabatabai S., Greve M.H. (2019). Mapping soil organic matter contents at field level with Cubist, Random Forest and kriging. Geoderma.

[39] Rial M., Martínez Cortizas A., Rodríguez-Lado L. (2017). Understanding the spatial distribution of factors controlling topsoil organic carbon content in European soils. Sci. Total Environ..

[40] Vaysse K., Lagacherie P. (2017). Using quantile regression forest to estimate uncertainty of digital soil mapping products. Geoderma.

[41] Boubehziz S., Khanchoul K., Benslama M., Benslama A., Marchetti A., Francaviglia R., Piccini C. (2020). Predictive mapping of soil organic carbon in Northeast Algeria. Catena.

[42] Sindayihebura A., Ottoy S., Dondeyne S., Van Meirvenne M., Van Orshoven J. (2017). Comparing digital soil mapping techniques for organic carbon and clay content: Case study in Burundi’s central plateaus. Catena.

[43] Xu Y., Smith S.E., Grunwald S., Abd-Elrahman A., Wani S.P., Nair V.D. (2018). Estimating soil total nitrogen in smallholder farm settings using remote sensing spectral indices and regression kriging. Catena.

[44] Arrouays D., Grundy M.G., Hartemink A.E., Hempel J.W., Heuvelink G.B.M., Hong S.Y., Lagacherie P., Lelyk G., McBratney A.B., McKenzie N.J., Sparks D.L. (2014). Chapter Three—GlobalSoilMap: Toward a Fine-Resolution Global Grid of Soil Properties. Advances in Agronomy.

[45] Malone B.P., McBratney A.B., Minasny B. (2011). Empirical estimates of uncertainty for mapping continuous depth functions of soil attributes. Geoderma.

[46] Deng X., Chen X., Ma W., Ren Z., Zhang M., Grieneisen M.L., Long W., Ni Z., Zhan Y., Lv X. (2018). Baseline map of organic carbon stock in farmland topsoil in East China. Agric. Ecosyst. Environ..

[47] Deng X., Ma W., Ren Z., Zhang M., Grieneisen M.L., Chen X., Fei X., Qin F., Zhan Y., Lv X. (2020). Spatial and temporal trends of soil total nitrogen and C/N ratio for croplands of East China. Geoderma.

[48] Li X., Shang B., Wang D., Wang Z., Wen X., Kang Y. (2020). Mapping soil organic carbon and total nitrogen in croplands of the Corn Belt of Northeast China based on geographically weighted regression kriging model. Comput. Geosci..

[49] Wang S., Zhuang Q., Wang Q., Jin X., Han C. (2017). Mapping stocks of soil organic carbon and soil total nitrogen in Liaoning Province of China. Geoderma.

[50] Wang S., Adhikari K., Wang Q., Jin X., Li H. (2018). Role of environmental variables in the spatial distribution of soil carbon (C), nitrogen (N), and C:N ratio from the northeastern coastal agroecosystems in China. Ecol. Indic..

[51] Aula L., Macnack N., Omara P., Mullock J., Raun W. (2016). Effect of Fertilizer Nitrogen (N) on Soil Organic Carbon, Total N, and Soil pH in Long-Term Continuous Winter Wheat (*Triticum Aestivum* L.). Commun. Soil Sci. Plant Anal..

[52] Sun X.-L., Minasny B., Wu Y.-J., Wang H.-L., Fan X.-H., Zhang G.-L. (2023). Soil organic carbon content increase in the east and south of China is accompanied by soil acidification. Sci. Total Environ..

[53] Wu Z., Sun X., Sun Y., Yan J., Zhao Y., Chen J. (2022). Soil acidification and factors controlling topsoil pH shift of cropland in central China from 2008 to 2018. Geoderma.

[54] Zhang X., Guo J., Vogt R.D., Mulder J., Wang Y., Qian C., Wang J., Zhang X. (2020). Soil acidification as an additional driver to organic carbon accumulation in major Chinese croplands. Geoderma.

[55] Liu Y., Gao P., Zhang L.Y., Niu X., Wang B. (2016). Spatial heterogeneity distribution of soil total nitrogen and total phosphorus in the Yaoxiang watershed in a hilly area of northern China based on geographic information system and geostatistics. Ecol. Evol..

[56] Wadoux A.M.J.C. (2019). Using deep learning for multivariate mapping of soil with quantified uncertainty. Geoderma.

[57] Costa E.M., Tassinari W.d.S., Pinheiro H.S.K., Beutler S.J., dos Anjos L.H.C. (2018). Mapping Soil Organic Carbon and Organic Matter Fractions by Geographically Weighted Regression. J. Environ. Qual..

[58] Wang K., Zhang C., Li W. (2013). Predictive mapping of soil total nitrogen at a regional scale: A comparison between geographically weighted regression and cokriging. Appl. Geogr..

[59] Zhu Q., Lin H.S. (2010). Comparing Ordinary Kriging and Regression Kriging for Soil Properties in Contrasting Landscapes. Pedosphere.

[60] Kuhn M., Wing J., Weston S., Williams A., Keefer C., Engelhardt A., Cooper T., Mayer Z., Kenkel B., Team R.C. (2020). Package ‘caret’. R J..

[61] Team R.C. (2020). A Language and Environment for Statistical Computing.

[62] Xiong X., Grunwald S., Myers D.B., Kim J., Harris W.G., Comerford N.B. (2014). Holistic environmental soil-landscape modeling of soil organic carbon. Environ. Model. Softw..

[63] Kursa M.B., Rudnicki W.R. (2010). Feature Selection with the Boruta Package. J. Stat. Softw..

[64] Breiman L. (2001). Random Forests. Mach. Learn..

[65] Biau G., Scornet E. (2016). A random forest guided tour. TEST.

[66] Lopes M.E. (2019). Estimating the algorithmic variance of randomized ensembles via the bootstrap. Ann. Stat..

[67] Liaw A., Wiener M. (2018). Package ‘randomForest’: Breiman and Cutler’s Random Forests for Classification and Regression.

[68] Mitran T., Mishra U., Lal R., Ravisankar T., Sreenivas K. (2018). Spatial distribution of soil carbon stocks in a semi-arid region of India. Geoderma Reg..

[69] Meinshausen N. (2006). Quantile regression forests. J. Mach. Learn. Res..

